# Associations of mood symptoms with NYHA functional classes in angina pectoris patients: a cross-sectional study

**DOI:** 10.1186/s12888-019-2061-3

**Published:** 2019-03-05

**Authors:** Han Yin, Yuting Liu, Huan Ma, Guihao Liu, Lan Guo, Qingshan Geng

**Affiliations:** 1 Department of Cardiology, Guangdong Cardiovascular Institute, Guangdong Provincial People’s Hospital, Guangdong Academy of Medical Sciences, No.106 Zhongshan Er Road, Guangzhou, 510080 People’s Republic of China; 2 School of Medicine, South China University of Technology, Guangzhou, China; 3 Department of Cardiac Rehabilitation, Guangdong Cardiovascular Institute, Guangdong Provincial People’s Hospital, Guangdong Academy of Medical Sciences, Guangzhou, China; 4 Department of Epidemiology, Guangdong Cardiovascular Institute, Guangdong Provincial People’s Hospital, Guangdong Academy of Medical Sciences, Guangzhou, China

**Keywords:** Depression, Anxiety, Angina pectoris, New York heart association class

## Abstract

**Background:**

Depression and anxiety are prevalent and associated with a worse prognosis in coronary heart disease (CHD) patients. However, the influence of disease severity on mood symptoms is unknown. The specific associations of mood symptoms with NYHA classes remain unexplored.

**Methods:**

In this cross-sectional study, 443 consecutive inpatients with angina pectoris (AP) confirmed by angiography were included into analysis. Somatic and cognitive symptom scores derived from Patient Health Questionnaire (PHQ-9) and Generalized Anxiety Disorder Scale (GAD-7) were used to assess mood symptoms. Predictors for depression and anxiety with strict and lax standards were compared. We hypothesized NYHA classification to be an indicator of disease severity through analyses with clinical features using ordinal logistic model. Applying both binary and ordinal logistic models, we evaluated the associations of mood symptoms with NYHA classes.

**Results:**

Discrepancy of disease severity existed between the depressed and nondepressed. NYHA classification was proved to be an integrated index under influence of age, coronary stenosis, heart failure and diabetes. NYHA class I and II individuals with AP were at equivalent risk for depression (NYHA II vs I: binary model OR 1.32 (0.59,2.96), *p* = 0.50; ordinal model OR 1.17 (0.73,1.88), *p* = 0.52), however NYHA class III/IV patients shared a sharply higher risk (NYHA III/IV vs I: binary model OR 3.32 (1.28,8.61), *p* = .013; ordinal model OR 3.94 (2.11,7.36), *p* < .001). Analyses on somatic and cognitive depressive symptoms confirmed this finding and hinted a greater impact of education background on mood when patient’s condition is unstable. Anxiety seemed in the whole picture irrelevant with NYHA classes. Comparing with NYHA class I/II, AP patients in NYHA class III/IV tended to be less anxious. However, when CHD became unstable, the calmness may immediately be broken up. A great distinction of the ratio of anxiety and depression symptom scores between NYHA class III/IV stable and unstable AP patients (*p* = .018) was observed.

**Conclusions:**

Mood symptoms in CHD patients are to a great extend derived from disease itself. Only for patients with relatively serious physical condition, unexpected discomforts caused by disease notably impact the emotions. Education background tends to influence the mood especially when disease is still unstable.

**Electronic supplementary material:**

The online version of this article (10.1186/s12888-019-2061-3) contains supplementary material, which is available to authorized users.

## Background

Depression and anxiety, more prevalent in CHD patients than the general population, are associated with an increased risk of worse prognosis [1–4]. However, these associations in many studies weaken or vanish when adjusted for variables that can reflect physical conditions [5–7], indicating a close correlation between emotional symptoms and disease severity [8–10]. Few researchers have particularly studied the alteration pattern of mood symptoms along with deterioration of CHD. Reasons for this phenomenon lie: (1) no explicit criteria exists for disease severity grading; (2) it seems a common sense for seriously ill patients to be in a bad state of mind. It is obvious that disease severity influences the clinical outcomes. Knowing the specific association of mood symptoms with disease severity may help to reach a better understanding of the impact of mood on prognosis, see through some confusing findings about anxiety and depression and find out the most efficient therapies for patients.

Searching through the articles, there are hardly any researches adjusting with same variables to eliminate the influence of disease severity on outcomes. New York Heart Association (NYHA) classification [11], as a widely used clinical tool which emphasizes the subjective cardiac symptoms on daily activity, possesses good predictive value of cardiopulmonary function [12, 13], physical status [14], quality of life [15] and clinical outcomes for example stroke [16], hospitalization [15] and mortality [15, 17]. We hypothesize NYHA class to be a simple but integrated indicator of physical status and can be utilized to assess the associations of mood symptoms with physical condition.

To fully understand the differences of emotional state under different physical condition, and under the background that several recent studies report that somatic rather than cognitive depressive symptoms correlate with lower heart rate variability [18] and predict worse long-term outcomes in CHD patients [19–22]. we split PHQ-9 into somatic and cognitive depressive symptoms based on confirmatory factor analysis and analyzed the correlation of depression, anxiety and their internal relations with NYHA classes in both stable angina pectoris (SAP) and unstable angina pectoris (UAP) patients. Through all these analyses, we hoped to reach a better understanding of mood symptoms in CHD patients and its change pattern along with worsening of physical condition. This may be of guiding significance for the timing of intervention and the selection of treatment.

## Methods

### Design

**Fig. 1:**
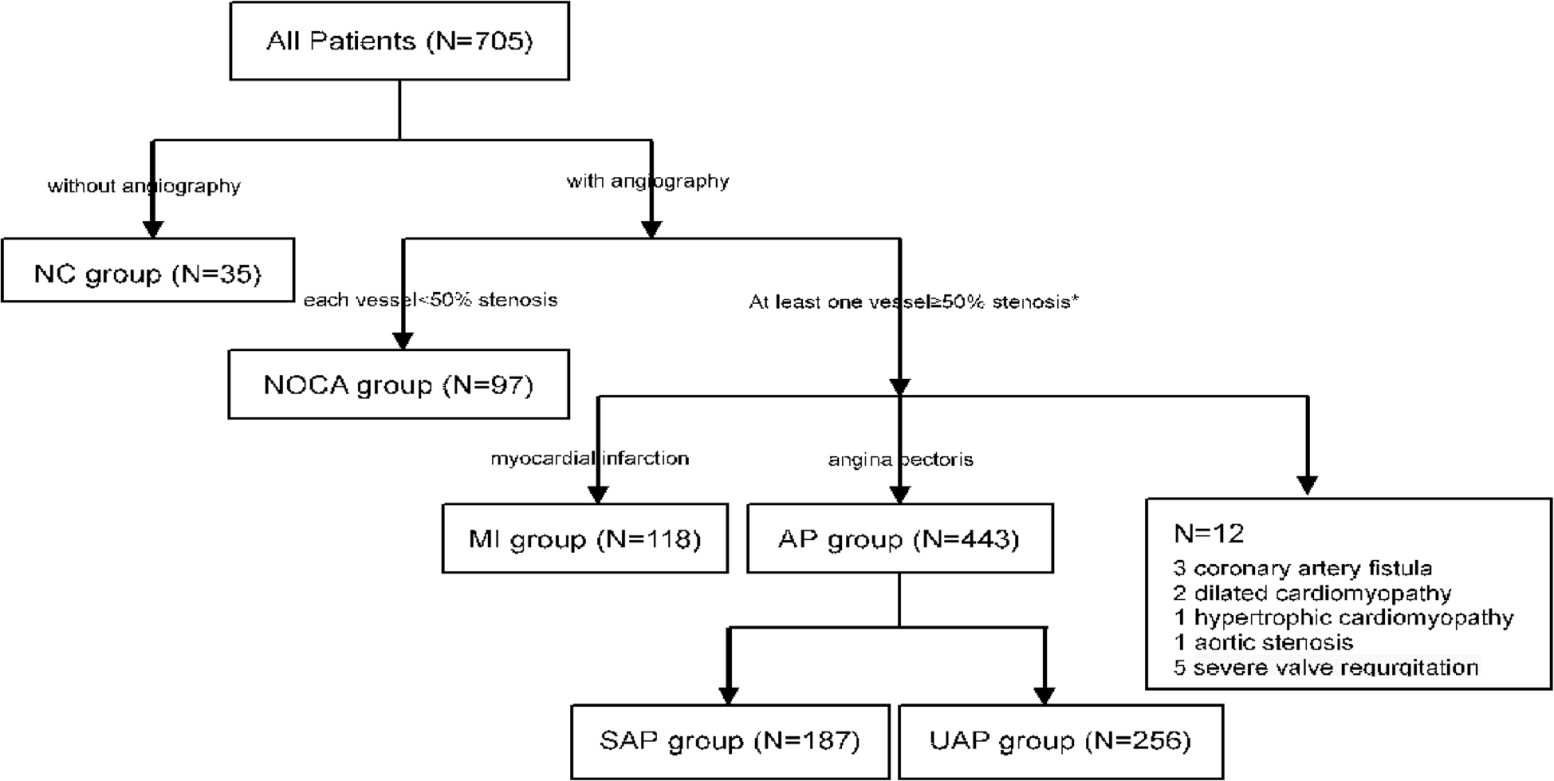
Categorization of inpatients surveyed in study. Abbreviation: NC: no coronary angiography; NOCA: no obstructive coronary artery; MI: myocardial infarction; AP: angina pectoris; SAP: stable angina pectoris; UAP: unstable angina pectoris. *: inpatients with at least one obstructive vessel (≥50%) confirmed by coronary angiography or with a history of coronary artery bypass grafting or coronary stent implantation were included

This is a cross-sectional study for investigating the discrepancies of mood symptoms of Chinese patients in different coronary condition and CHD subtypes and the determinants for depression and anxiety. 705 consecutive inpatients with primary diagnosis of CHD at admission in Guangdong Provincial People’s Hospital were surveyed between October 2017 and January 2018. Results of clinical tests and coronary angiography (CAG) as well as discharge diagnosis were acquired from medical records to ensure the correct patient categorization (Fig. 1). Chinese version of PHQ-9, GAD-7 and a self-designed short questionnaire about valuable information were applied. All participants were surveyed in comparatively stable condition and under supervision of one well-trained psycho-cardiologist, who was responsible for elucidating the PHQ-9 and GAD-7 questionnaires, assisting patients with failing eyesight or low literacy and conducting a concise review to guarantee data accuracy.

### Patients selection

The current paper concerns a cross-sectional analysis of the baseline status of the angina pectoris inpatients. Inpatients with main discharge diagnosis of angina pectoris and a history of coronary artery bypass grafting or coronary stent implantation or with at least one narrow epicardial coronary artery (≥50%) confirmed by CAG during this hospitalization were included. Participants with severe valvular heart disease, or severe cardiomyopathy unlikely caused by coronary stenosis, or other complications that might interfere the mechanism that symptoms were primarily resulted from the narrowed coronary were excluded, leaving a sample of 443 subjects (187 SAP and 256 UAP according to Braunwald criteria [23]) into analysis (Fig. 1). The study was approved by the Medical Ethics Committee of Guangdong Provincial People's Hospital. Written informed consent were obtained from all participants.

### New York heart association classification

NYHA classification [11] is a widely used clinical tool that measures the cardiac functional capacity. The assessment of NYHA class was mainly based on the medical records at admission. However, for the missing data, two cardiologists separately estimated the NYHA class and discussed with a third doctor if inconsistence appeared. To unify the criteria, we defined that conditions triggering fatigue, palpitation, dyspnea, or anginal pain of NYHA class III patients were walking 20–100 m or climbing one flight of stairs at normal pace.

### Patient health questionnaire – 9

The PHQ-9 is a valid screening tool for depression in accordance with DSM-IV criteria for major depressive disorder (MDD) [24, 25]. The 9 items which evaluate the depression symptoms are rated on a 0–3 Likert-type scale with higher score on each item representing more frequently being bothered by the symptom in the last 2 weeks. It has been demonstrated to be a reliable predictor of depression severity with mild, moderate, moderately severe to severe depression corresponding to a score of 5, 10 and 15, respectively [24]. PHQ-9 score ≥ 10 indicates clinical depression and has a sensitivity of 88% and a specificity of 88% for major depression [25]. Given the fact that even mild depression symptom classified by PHQ-9 is associated with a worse prognosis of cardiac patients [26], clinical characteristics between subjects with PHQ-9 score < 5 and ≥ 5 have also been compared. The Chinese version of PHQ-9 has been validated in Chinese cardiac patients [27].

### Somatic and cognitive depressive symptoms

A number of researches have proved that PHQ-9 has two-factor structure and can be divided into somatic and cognitive depression symptom subscales. To be accurate, we listed 5 representative models (one-factor model: Model 1 [28]; four two-factor models: Model 2a [29–31], Model 2b [18, 19, 21], Model 2c [32–34], Model 2d [30]) (see Additional file 1: Table S1), and implemented confirmatory factor analysis (CFA) with items as continuously-scaled and maximum likelihood estimation with a mean-adjustment analysis method for non-normality data using Mplus 7 software. Model 2c turned out to be the best model with fit indices indicating adequately fit [35]. Internal consistency coefficients (Cronbach’s α) were 0.76 for factor 1 (somatic) and 0.79 for factor 2 (cognitive). The error-free factors were correlated at 0.85. We accordingly calculated the sum scores of the two dimensions as factor scores (somatic and cognitive).

### Generalized anxiety disorder scale– 7

GAD-7 is a 7-item self-report scale based on DSM-IV criteria [36]. Items of GAD-7 are also rated on a 0–3 Likert-type scale. It measures the severity of generalized anxiety disorders and also exhibits good convergent validity when compared with other commonly-used anxiety scales [37]. Total score ranges from 0 to 21 with a score of 0–4, 5–9, 10–14, 15–21 representing normal, mild, moderate and severe levels of anxiety, respectively. Analogously, GAD-7 score ≥ 10 indicates clinical anxiety, since the sensitivity and specificity for generalized anxiety disorder reached 89 and 82%, respectively. The minimal clinical important difference has not been established. Considering the widely use of the cutoff of 5 to distinguish patients from normal state, differences between patients with GAD-7 score < 5 and ≥ 5 have also been compared. The Chinese version of GAD-7 has been validated in Chinese cardiac patients [38]. Previous studies have shown the underlying structure of GAD-7 to be unidimension [37, 39].

### Coronary artery stenosis severity, education background and creatinine clearance

Coronary artery stenosis severity was assessed according to the number of three main vessels with stenosis ≥50% as shown by angiography. However, a ≥ 30% lumen stenosis in left main coronary artery would be directly classified as the highest level of severity.

Due to the difference of education systems, the schooling year for participants in each stage may not be the same. The four levels of education background represented illiteracy or primary school, junior high school, senior high school and technical school or college or university level.

Creatinine clearance was estimated using the Cockcroft-Gault formula with the value of serum creatinine tested at admission.

### Statistical analysis

Statistical analysis mainly contained three parts: (1) We first compared patients’ characteristics according to different cutoffs for depression and anxiety to figure out the dominant predictors of mood disturbance; (2) Next, we evaluated whether NYHA classification could be an integrated index indicating patients’ status; (3) Finally, we explored the association between NYHA classes and depression or anxiety symptoms.

**·Part 1** Clinical characteristics were compared between patients with questionnaire score < 10 and ≥ 10 as well as < 5 and ≥ 5 with Student’s t-test and Wilcoxon rank-sum test for continuous variables and Chi-square or Fisher’s Exact test or Cochran-Mantel-Haenszel test for categorical variables.

**·Part 2** Since only 6 patients were categorized as NYHA IV, we incorporated NYHA III and NYHA IV and compared clinical characteristics between the 3 groups using one-way analysis of variance (ANOVA) or chi-square tests or Kruskal-Wallis test. We chose to model NYHA classes (I, II, III/IV) as ordinal outcomes using ordinal logistic regression, adjusting for all physical condition variables with a significant association in univariate analyses along with age, sex, body mass index and education background.

**·Part 3** We conducted ordinal logistic regression analyses taking into account all depression and anxiety severities as ordinal outcomes (0, 1, 2, 3) and compared the results with binary logistic regression models, which treated questionnaire scores as dichotomous outcome variables with the cutoff point of 10. Analogous analyses were also made with somatic and cognitive depressive symptom scores through converting them to dichotomous variables depending on whether the upper quartile was reached. All models were adjusted for age, sex, body mass index (BMI) and education background. The correlation between PHQ-9 and GAD-7 scores was assessed using Pearson’s correlation coefficients. Their internal relation of AP, SAP and UAP patients in different NYHA classes were analyzed with linear regression model and plotted with R software (version 3.5.1). Ratio of anxiety and depression symptom score between SAP and UAP groups was compared using Student’s t-test.

Except for data of Nt-ProBNP and LVEF, model independent variates were missing for at least 1 study variate in 14 patients (3.2%), with no study variate having > 1.8% missing data. Mean or median imputation depending on distribution pattern were applied using SAS STDIZE procedure. All tests for significance were two-tailed at the threshold of 0.05 and were performed with SAS 9.4 software.

## Results

### Predictors of elevated depression and anxiety symptoms

Of the 443 consecutive angina pectoris inpatients screened, 123(27.8%), 34(7.7%) and 15(3.4%) inpatients were categorized as with mild, moderate and moderately severe to severe depression symptom, 103(23.3%), 13(2.9%), 11(2.5%) with mild, moderate and severe anxiety symptom. Patients’ characteristics were presented in Table 1 and Additional file 2: Table S2.

**Table 1:** Characteristics of patients stratified by depression severity

Variables	Total	Non-depressed	depressed		*p* value	p value
			mild dep.	mod-severe dep.		
	*N* = 443	*N* = 271	*N* = 123	*N* = 49	not clinical vs clinical	nondepressed vs depressed
		61.2% (score < 5)	27.8%	11.1% (score ≥ 10)		
Demographics						
Age,mean ± SD,y	63.9 ± 9.8	63.0 ± 9.5	65.5 ± 10.0	64.9 ± 10.8	0.47	.019
Male,No.(%)	337(76.1)	223(82.3)	79(64.2)	35(71.4)	0.42	<.001
Body mass index,mean ± SD,kg/m2	24.5 ± 3.1	24.6 ± 2.7	24.4 ± 3.5	24.2 ± 3.7	0.51	0.41
Clinical characteristics						
NYHA class I-IV,No.(%)					.017	<.001
Class I	115(26.0)	79(29.2)	27(22.0)	9(18.4)		
Class II	261(58.9)	169(62.4)	66(53.7)	26(53.1)		
Class III-IV	67(15.1)	23(8.5)	30(24.4)	14(28.6)		
Ejection Fraction,mean ± SD,%	59.3 ± 11.0	60.3 ± 9.9 *N* = 238	56.4 ± 12.3 *N* = 112	60.8 ± 12.0 *N* = 43	0.33	.024
Nt-ProBNP,median(interquartile),pg/mL	123(50–382)	106(48–309) *N* = 231	180(72–721) *N* = 99	124(47–341) *N* = 39	0.98	.013
Creatinine Clearance,mean ± SD,ml/min	65.5 ± 21.7	68.4 ± 20.1	61.1 ± 22.4	60.7 ± 26.3	0.17	<.001
Type of angina pectoris,No.(%)					0.92	0.94
Unstable angina pectoris	256(57.8)	157(57.9)	71(57.7)	28(57.1)		
Stable angina pectoris	187(42.2)	114(42.1)	52(42.3)	21(42.9)		
Severity of coronary stenosis,No.(%)					0.62	0.34
1	93(21.0)	51(18.8)	33(26.8)	9(18.4)		
2	82(18.5)	54(19.9)	19(15.4)	9(18.4)		
3	268(60.5)	166(61.3)	71(57.7)	31(63.3)		
Social factors						
Education,No.(%)					.041	<.001
less than 6 years	115(26.0)	50(18.5)	44(35.8)	21(42.9)		
7–9 years	126(28.4)	85(31.4)	31(25.2)	10(20.4)		
10–12 years	97(21.9)	63(23.2)	25(20.3)	9(18.4)		
more than 12 years	105(23.7)	73(26.9)	23(18.7)	9(18.4)		
Marriage,No.(%)					.068	.023
Married	412(93.0)	258(95.2)	112(91.1)	42(85.7)		
Divorced or Widowed or Single	31(7.0)	13(4.8)	11(8.9)	7(14.3)		
Medical history,No.(%)						
Hypertension	276(62.3)	162(59.8)	78(63.4)	34(69.4)	0.25	0.26
Diabetes mellitus	154(34.8)	83(30.6)	52(42.3)	19(38.8)	0.53	.022
Prior PCI	167(37.7)	102(37.6)	43(35.0)	22(44.9)	0.27	0.97
History of antidepressant treatment	17(3.8)	4(1.5)	6(4.9)	7(14.3)	<.001	.001
Medication use,No.(%)						
ACEI or ARB	317(71.6)	190(70.1)	93(75.6)	34(69.4)	0.72	0.40
β blocker	384(86.7)	230(84.9)	109(88.6)	45(91.8)	0.26	0.16
Mono antipletelet therapy	63(14.2)	38(14.0)	19(15.4)	6(12.2)	0.67	0.88
Dual antiplatelet therapy	370(83.5)	228(84.1)	100(81.3)	42(85.7)	0.66	0.66
Statin	430(97.1)	266(98.2)	120(97.6)	47(95.9)	0.36	0.46
Aldosterone receptor antagonist	42(9.5)	16(5.9)	18(14.6)	8(16.3)	.083	.001
Loop diuretic	47(10.6)	18(6.6)	20(16.3)	9(18.4)	.062	<.001
Anticoagulant	20(4.5)	10(3.7)	8(6.5)	2(4.1)	> 0.99	0.29
Antidepressant	9(2.0)	4(1.5)	0(0)	5(10.2)	.001	0.49
Somatic symptom score,mean(SD)	3.05(2.87)	1.39(1.18)	4.37(1.44)	8.90(2.81)	<.001	<.001
Cognitive symptom score,mean(SD)	1.36(1.98)	0.39(0.63)	1.98(1.46)	5.16(2.73)	<.001	<.001
Somatic / Dep. symptom score,%	69.2	78.1	68.9	63.3		

Note: Clinical characteristics were compared between subjects with PHQ-9 score < 10 and ≥ 10 (not clinical vs clinical) as well as < 5 and ≥ 5 (non-depressed vs depressed)

Abbreviation: dep.: depression; mod-severe dep.: moderate or severe depression; PCI: percutaneous transluminal coronary intervention; ACEI: angiotensin converting enzyme inhibitor; ARB: angiotensin receptor blocker

Compared with individual who had no or mild depression symptom, those with clinical depression (PHQ-9 score ≥ 10) were more likely to be less educated (*p* = .041), with higher NYHA classes (*p* = .017) and a history of antidepressant treatment (*p* < .001). A slight trend toward significance was observed for a prescription of loop diuretics (*p* = .062) or aldosterone receptor antagonist (*p* = .083). However, when comparing those not depressed with depressed patients, features that marked worse physical status became quite outstanding (see Table 1). Besides, the depressed participants tended to be older (*p* = .019), female (*p* < .001), without marriage partner (*p* = .023) and less educated (*p* < .001). The average scores of somatic depressive depression symptoms in each depression severity groups were 1.39 (SD 1.18), 4.37 (SD 1.44), 8.90 (SD 2.81), taking up 78.1, 68.9 and 63.3% of the total score, respectively.

Unlike depression, no difference except for an antidepressant treatment history (*p* = .010) was observed between the patients with or without clinical anxiety. Interestingly in comparison of the anxious and non-anxious, we noticed that anxious subjects tended to be female (*p* < .001), less educated (*p* = .001), with less severe coronary artery stenosis (*p* = .050) and a history of antidepressant treatment (*p* < .001) (see Additional file 2: Table S2).

### NYHA classes and clinical characteristics

In univariate analyses (Table 2), we discovered that significant differences existed among groups in age (*p* < .001), EF (*p* < .001), Nt-ProBNP (p < .001), creatinine clearance (*p* < .001), coronary artery stenosis severity (*p* < .001), medical history of hypertension (*p* = .003) or diabetes (*p* < .001), prescription of loop diuretics (*p* < .001) or aldosterone receptor antagonist (*p* < .001), depression severity (*p* < .001), somatic (*p* < .001) and cognitive (*p* < .001) depressive symptoms, but not in anxiety severity (*p* = 0.99), type of AP (*p* = 0.24), nor education background (*p* = 0.83). After multivariate adjustment using ordinal logistic regression model, significance retained for age (*p* = .024), EF (*p* = .037), Nt-ProBNP (*p* = .006), coronary artery stenosis severity (*p* = .034) and history of diabetes (*p* = .016) (see Additional file 3: Table S3), revealing a multiple impact of age, CHD severity, diabetes and heart failure on NYHA classes.

**Table 2:** Characteristics of patients stratified by New York Heart Association functional class

Variables	Total	NYHA class I	NYHA class II	NYHA class III-IV	*p* value
	N = 443	*N* = 115	*N* = 261	*N* = 67	
		26.0%	58.9%	15.1%	
Characteristic of patients					
Age,mean ± SD,y	63.9 ± 9.8	61.3 ± 9.3	64.0 ± 9.8	68.2 ± 9.5	<.001
Male,sex,No.(%)	337(76.1)	85(73.9)	199(76.2)	53(79.1)	0.73
Body mass index,mean ± SD,kg/m^2^	24.5 ± 3.1	24.5±	24.5 ± 3.0	24.6 ± 3.6	0.94
Ejection Fraction,mean ± SD,%	59.3 ± 11.0	61.0 ± 8.2 *N* = 100	60.8 ± 12.3 *N* = 235	50.1 ± 16.1 *N* = 58	<.001
Nt-ProBNP,median(interquartile),pg/mL	123(50–382)	88(37–234) *N* = 92	118(50–309) *N* = 228	733(133–3892) N = 49	<.001
Creatinine Clearance,mean ± SD,ml/min	65.5 ± 21.7	69.5 ± 18.2	67.0 ± 22.2	53.0 ± 21.0	<.001
Type of angina pectoris,No.(%)					0.24
Unstable angina pectoris	256(57.8)	65(56.5)	158(60.5)	33(49.3)	
Stable angina pectoris	187(42.2)	50(43.5)	103(39.5)	34(50.7)	
Severity of coronary artery stenosis,No.(%)					<.001
1	93(21.0)	40(34.8)	46(17.6)	7(10.4)	
2	82(18.5)	19(16.5)	54(20.7)	9(13.4)	
3	268(60.5)	56(48.7)	161(61.7)	51(76.1)	
Mood symptoms					
Depression severity,No.(%)					<.001
Non-depressed	271(61.2)	79(68.7)	169(64.8)	23(34.3)	
Mild depression symptom	123(27.8)	27(23.5)	66(25.3)	30(44.8)	
Moderate or severe depression symptom	49(11.1)	9(7.8)	26(10.0)	14(20.9)	
Somatic depressive symptom score,mean(SD)	3.05(2.87)	2.81(2.64)	2.79(2.76)	4.48(3.28)	<.001
Cognitive depressive symptom score,mean(SD)	1.36(1.98)	1.21(1.94)	1.20(1.76)	2.24(2.58)	<.001
Anxiety severity,No.(%)					0.99
Non-anxious	316(71.3)	80(69.6)	186(71.3)	50(74.6)	
Mild anxiety symptom	103(23.3)	30(26.1)	62(23.8)	11(16.4)	
Moderate or severe anxiety symptom	24(5.4)	5(4.3)	13(5.0)	6(9.0)	
Social economic factors					
Education,No.(%)					0.83
less than 6 years	115(26.0)	26(22.6)	69(26.4)	20(29.9)	
7–9 years	126(28.4)	33(28.7)	75(28.7)	18(26.9)	
10–12 years	97(21.9)	29(25.2)	52(19.9)	16(23.9)	
more than 12 years	105(23.7)	27(23.5)	65(24.9)	13(19.4)	
Marriage,No.(%)					0.57
Married	412(93.0)	109(94.8)	240(92.0)	63(94.0)	
Divorced or Widowed or Single	31(7.0)	6(5.2)	21(8.0)	4(6.0)	
Medical history,No.(%)					
Hypertension	276(62.3)	56(48.7)	173(44.8)	45(67.2)	.003
Diabetes mellitus	154(34.8)	28(24.3)	90(34.5)	36(53.7)	<.001
Prior PCI	167(37.7)	42(36.5)	93(35.6)	34(50.7)	.069
History of antidepressant treatment	17(3.8)	4(3.5)	9(3.4)	4(6.0)	0.61
Medication use,No.(%)					
ACEI or ARB	317(71.6)	76(66.1)	192(73.6)	49(73.1)	0.32
β blocker	384(86.7)	95(82.6)	232(88.9)	57(85.1)	0.23
Aldosterone receptor antagonist	42(9.5)	2(1.7)	14(5.4)	26(38.8)	<.001
Loop diuretic	47(10.6)	2(1.7)	16(6.1)	29(43.3)	<.001
Anticoagulant	20(4.5)	3(2.6)	12(4.6)	5(7.5)	0.31
Antidepressant	9(2.0)	1(0.9)	6(2.3)	2(3.0)	0.55

Abbreviation: PCI: percutaneous transluminal coronary intervention; ACEI: angiotensin converting enzyme inhibitor; ARB: angiotensin receptor blocker

### Associations of NYHA classes with depression and anxiety

**Fig. 2:**
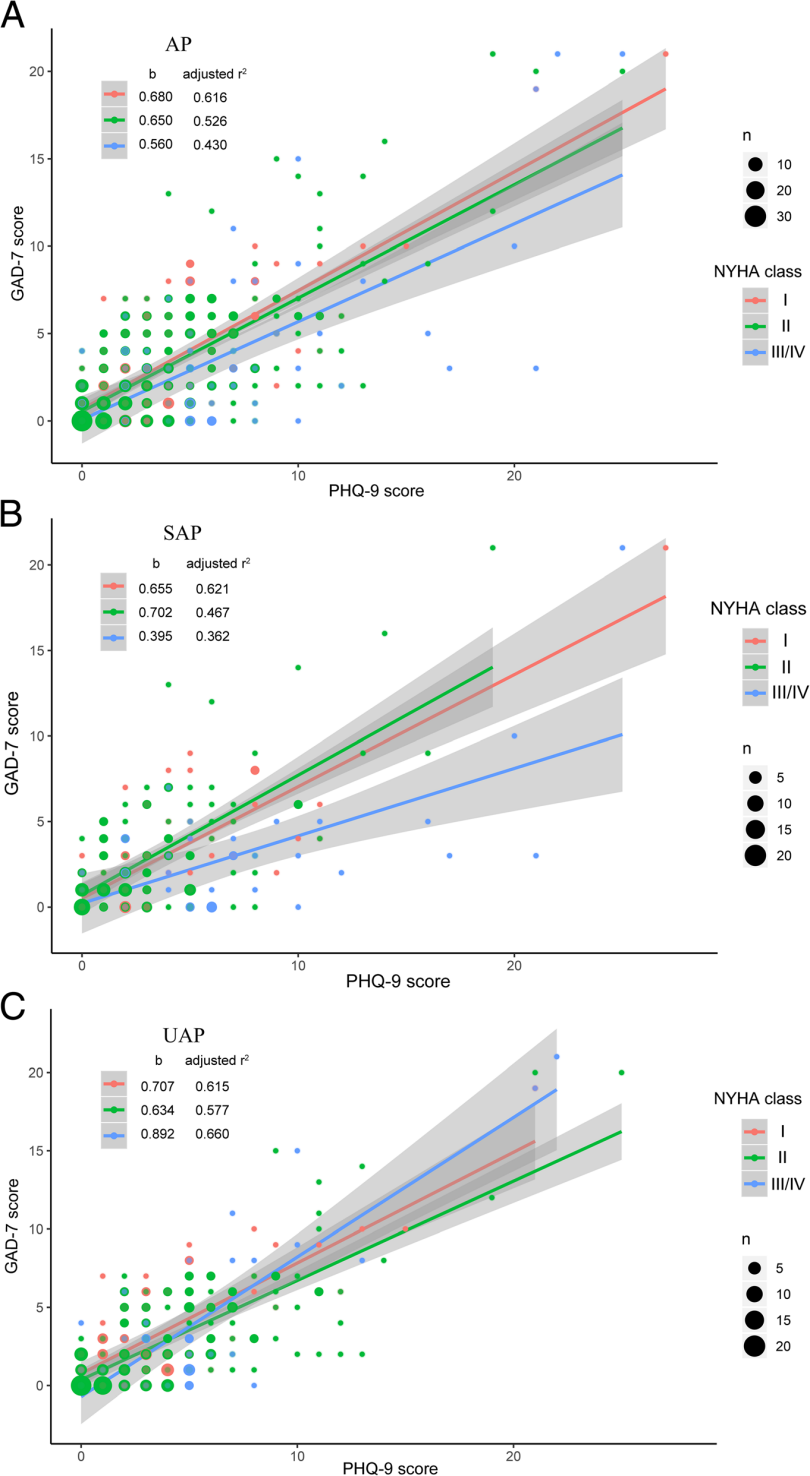
Linear regression analyses of correlations between anxiety and depression symptoms of (**a**) angina pectoris, (**b**) stable angina pectoris, and (**c**) unstable angina pectoris patients in different NYHA classes. Note: The correlations of depression and anxiety in total angina pectoris patients and unstable angina pectoris patients under different NYHA classes were non-differential. However, stable angina pectoris participants in NYHA III/IV seemed to be less anxious under the same level of depression

The Pearson’s correlation coefficients of PHQ-9 and GAD-7 scores was 0.72 (*p* < .001). As shown in Fig. 2, a non-differential interrelationship of depression and anxiety in different NYHA classes in AP (Fig. 2.A) and UPA patients (Fig. 2.C) was observed. For SAP, subjects in NYHA class III/IV seemed to be less anxious than those in NYHA class I and II under the same level of depression (Fig. 2.B). The ratio of anxiety and depression symptom scores differed significantly between SAP and UAP patients in NYHA class III/IV with at least mild depression symptoms (*p* = .018), but no difference between groups exited in separate analyses neither for anxiety nor depression.

Comparing the results of the binary and ordinal logistic models (see Table 3), a great consistency was observed in analyses for depression and anxiety in SAP patients, but not in UAP counterparts. For SAP patients, NYHA classes was significantly associated with levels of depression (binary model: *p* = .010; ordinal model: *p* < .001). This close correlation was also verified in UAP patients though only with ordinal model (binary model: *p* = 0.46; ordinal model: *p* = .005). Detailed analyses demonstrated that NYHA class I and II subjects in all AP types were statistically at equivalent risk for depression (for AP: NYHA II vs I binary model OR 1.32 (0.59,2.96), *p* = 0.50; ordinal model OR 1.17 (0.73,1.88), *p* = 0.52), although NYHA II subjects with UAP seemed more likely to be depressed in comparison with those SAP counterparts through the results of both models and analyses of somatic and cognitive depressive symptoms. One possible reason for this phenomenon is that SAP patients in NYHA class II may more frequently have the psychological expectancy of angina when doing excessive activities, but NYHA class I patients may not. NYHA III/IV patients, by contrast, shared a sharply higher risk (for AP: NYHA III/IV vs I binary model OR 3.32 (1.28,8.61), *p* = .013; ordinal model OR 3.94 (2.11,7.36), *p* < .001). NYHA class was found to be not associated with levels of anxiety regardless of the AP types. Additionally, education background was demonstrated to correlate with the risk for depression and anxiety only in UAP inpatients.

**Table 3:** Associations of depression and anxiety with NYHA classes using binary and ordinal logistic regression models

	NYHA classes					Gender		Age		Education						
	NYHA class II vs I		NYHA class III /IV vs I		overall	female vs male		per 1 year increase		7-9 vs less than 6 years		10-12 vs less than 6 years		more then 12 vs less than 6 years		overall
	Odds Ratio (95% CI)	*p* value	Odds Ratio (95% CI)	*p* value	*p* value	Odds Ratio (95% CI)	*p* value	Odds Ratio (95% CI)	*p* value	Odds Ratio (95% CI)	*p* value	Odds Ratio (95% CI)	*p* value	Odds Ratio (95% CI)	*p* value	*p* value
Stable angina pectoris																
depression as dichotomous variable	0.98 (0.28,3.47)	0.98	5.01 (1.30,19.3)	.019	.010	1.48 (0.47,4.67)	0.51	0.98 (0.93,1.04)	0.56	0.51(0.12,2.15)	0.36	1.25(0.31,5.07)	0.75	0.93(0.23,3.83)	0.92	0.64
as ordinal variable	0.84 (0.41,1.71)	0.63	4.36 (1.77,10.7)	.001	<.001	2.27 (1.11,4.66)	0.030	1.00 (0.97,1.04)	0.97	0.62(0.26,1.49)	0.29	1.30(0.52,3.24)	0.58	0.98(0.40,2.38)	0.97	0.41
Unstable angina pectoris																
depression as dichotomous variable	1.52 (0.52,4.47)	0.44	2.49 (0.60,10.4)	0.21	0.46	0.87 (0.33,2.27)	0.78	0.99 (0.94,1.04)	0.60	0.30(0.10,0.88)	.029	0.19(0.05,0.75)	.017	0.27(0.08,0.93)	.038	.026
as ordinal variable	1.61 (0.84,3.10)	0.15	4.39 (1.77,10.9)	.001	.005	1.48 (0.80.2.74)	0.21	0.99 (0.97,1.02)	0.68	0.32(0.16,0.64)	.001	0.26(0.12,0.56)	<.001	0.25(0.11,0.55)	<.001	<.001
Angina pectoris																
depression as dichotomous variable	1.32 (0.59,2.96)	0.50	3.32 (1.28,8.61)	.013	.021	1.06 (0.52,2.19)	0.87	0.99 (0.95,1.02)	0.45	0.38(0.16,0.87)	.023	0.43(0.18,1.06)	.065	0.43(0.18,1.04)	.060	.074
as ordinal variable	1.17 (0.73,1.88)	0.52	3.94 (2.11,7.36)	<.001	<.001	1.73 (1.09,2.75)	.019	1.00 (0.98,1.02)	0.83	0.43(0.25,0.72)	.002	0.50(0.28,0.88)	.016	0.44(0.25,0.79)	.005	.006
Stable angina pectoris																
anxiety as dichotomous variable	2.59 (0.29,22.9)	0.39	3.59 (0.30,43.4)	0.32	0.59	1.21 (0.23,6.41)	0.82	0.98 (0.90,1.06)	0.56	#						
as ordinal variable	1.13 (0.52,2.44)	0.76	0.80 (0.28,2.33)	0.69	0.77	1.71 (0.82,3.59)	0.16	1.00 (0.96,1.04)	>0.99	#						
Unstable angina pectoris																
anxiety as dichotomous variable	0.85 (0.24,3.01)	0.80	2.27 (0.47,10.9)	0.31	0.36	1.08 (0.29,3.97)	0.91	0.98 (0.93,1.04)	0.55	0.82(0.22,3.07)	0.76	0.39(0.07,2.22)	0.29	0.62(0.13,2.89)	0.54	0.74
as ordinal variable	0.98 (0.51,1.88)	0.94	1.37 (0.51,3.66)	0.53	0.74	2.27 (1.19,4.33)	.013	0.96 (0.93,0.99)	.014	0.50(0.25,1.02)	.058	0.45(0.20,0.99)	.047	0.17(0.06,0.46)	<.001	.005
Angina pectoris																
anxiety as dichotomous variable	1.21 (0.41,3.53)	0.73	2.39 (0.66,8.73)	0.19	0.35	0.93 (0.33,2.63)	0.88	0.97 (0.93,1.02)	0.28	0.37(0.12,1.20)	0.10	0.38(0.11,1.35)	0.13	0.46(0.14,1.53)	0.21	0.29
as ordinal variable	0.99 (0.60,1.92)	0.97	0.96 (0.47,1.95)	0.91	0.99	1.93 (1.18,3.16)	.008	0.98 (0.95,1.00)	.050	0.50(0.29,0.88)	.017	0.56(0.30,1.03)	.063	0.36(0.19,0.69)	.002	.013

*Abbreviation: NYHA class* New York Heart Association functional class, *BMI* body mass index

#: due to the limitation of small sample size, binary and ordinal logistic regression models were only adjusted for age, sex, and BMI for patients with stable angina pectoris

Similar trend was also revealed in binary logistic analyses for somatic and cognitive depressive symptoms as shown in Table 4. The only difference beyond their synchronous changes was that cognitive depressive symptoms in UAP and AP patients were affected by gender (for UAP patients: OR 2.11 (1.08,4.11), *p* = .029; for AP patients: OR 1.82 (1.10,3.01), *p* = .020), but somatic symptoms were not.

**Table 4:** Associations of somatic and cognitive depressive symptoms with NYHA classes using binary logistic regression model

	NYHA classes					Gender		Age		Education						
	NYHA class II vs I		NYHA class III/IV vs I		overall	female vs male		per 1 year increase		7-9 years vs less than 6		10-12 years vs less than 6		more than 12 vs less than 6		overall
	Odds Ratio (95% CI)	*p* value	Odds Ratio (95% CI)	*p* value	*p* value	Odds Ratio (95% CI)	*p* value	Odds Ratio (95% CI)	*p* value	Odds Ratio (95% CI)	*p* value	Odds Ratio (95% CI)	*p* value	Odds Ratio (95% CI)	*p* value	*p* value
Stable angina pectoris																
somatic depressive symptoms	0.61 (0.28,1.33)	0.22	3.41 (1.29,9.00)	.013	<.001	1.77 (0.79,3.93)	0.16	1.02 (0.98,,1.06)	0.43	0.81(0.31,2.11)	0.67	1.80 (0.65,4.95)	0.26	0.79 (0.29,2.15)	0.64	0.29
cognitive depressive symptoms	0.89 (0.40,1.97)	0.78	4.56 (1.69,12.3)	.003	<.001	1.64 (0.74,3.67)	0.23	1.00 (0.97,1.04)	0.88	0.46(0.17,1.24)	0.12	0.79 (0.28,2.19)	0.64	1.10 (0.43,2.87)	0.83	0.28
Unstable angina pectoris																
somatic depressive symptoms	1.41 (0.72,2.76)	0.32	2.80 (1.08,7.31)	.035	0.11	1.63 (0.85,3.11)	0.14	1.02 (0.99,1.05)	0.24	0.44(0.22,0.90)	.024	0.30 (0.13,0.69)	.004	0.32 (0.14,0.71)	.005	.009
cognitive depressive symptoms	1.23 (0.62,2.43)	0.55	2.77 (1.04,7.38)	.042	0.11	2.11 (1.08,4.11)	.029	0.97 (0.94,1.00)	.058	0.70(0.34,1.43)	0.32	0.33 (0.14,0.78)	.012	0.42 (0.18,0.99)	.048	.049
Angina pectoris																
somatic depressive symptoms	1.01 (0.61,1.66)	0.98	2.86 (1.47,5.56)	.002	.001	1.60 (0.98,2.61)	.060	1.02 (1.00,1.04)	0.12	0.55(0.32,0.96)	.035	0.60 (0.33,1.10)	.096	0.43 (0.23,0.79)	.007	.041
cognitive depressive symptoms	1.07 (0.64,1.78)	0.80	3.28 (1.67,6.44)	<.001	<.001	1.82 (1.10,3.01)	.020	0.98 (0.96,1.01)	0.14	0.61(0.35,1.08)	.090	0.45 (0.24,0.86)	.016	0.63 (0.34,1.17)	0.14	0.10

*Abbreviation: NYHA class* New York Heart Association functional class, *BMI* body mass index

## Discussion

In a sample of 443 AP inpatients, we compared patients’ characteristics according to different cutoffs for depression and anxiety and inferred that depression symptoms were aggravated along with the worsening of physical condition. Univariate analyses of NYHA classes with clinical characteristics further confirmed such inference. Next, though multivariate analysis we proved that NYHA classification could be an integrated index reflecting patients’ physical status. Finally, we explored the association between NYHA classes and depression or anxiety symptoms and concluded that only for patients with relatively serious physical condition, unexpected discomforts caused by disease notably impacted the emotions.

There has been a debate whether depression disorder in general population is the same thing as in the cardiac patients since long time ago. Our previous analyses of inpatients without or with coronary stenosis < 50% from the same cross-sectional study sample found the prevalence of clinical depression to be almost twice as high as the one in present study. With the findings mentioned above, it is reasonable to believe that “these two depression disorders” are not the same and may exist at the same time. Analysis of the ratio of somatic and cognitive symptom scores hinted a greater fluctuation of cognitive symptoms with the increase in depression severity. As a result, when cutoff point reached a certain value, the screening for depression becomes more dependent on cognitive symptoms. That is the reason why there is a difference in the results between using the cutoff point of 10 and 5, and why ordinal logistic model is more sensitive to physical condition than binary logistic model.

The rough correlation of mood state and NYHA class has been reported in univariate analyses of considerable previous studies [40–43]. However, in consideration of collinearity with other clinical features such as Pro-BNP, EF, creatinine and so on, few studies have treated NYHA classes as an integrated index reflecting disease severity and explored the associations with mood symptoms in multivariate regression models. Our finding was consistent with the expectation that angina pectoris patients in NYHA class III/IV compared to NYHA class I and II were at greater risk for depression.

In accord with the finding from Assari S. [44], our univariate analyses revealed that for AP patients, less coronary stenosis was associated with elevated anxiety symptoms. It seems that anxiety is more likely to be a stress response. Perhaps our body though constantly adjustment might have learned to “keep calm” in case of sympathetic activation or myocardial ischemia induced by mental stress [45] when with severe CHD. However, when it comes a stress exceeding the threshold physically or mentally, for example the loss of stability of CHD, the calmness may immediately be broken up.

Additionally, quite consistent with our common sense, it was discovered that education background engendered greater effect on mood symptoms in UAP patients. This might attribute to the differences in perception and anticipatory anxiety influenced by knowledge and the social support obtained from social status. In other words, this may indicate that patients in acute phase of CHD for example UAP or even AMI (acute myocardial infarction) can get more benefit from health education, or antidepressant therapy and psychological counseling. Several recent researches have indeed confirmed this hypothesis [46, 47].

To our knowledge, it is the first time that in one study the associations between NYHA classes and depression/anxiety in both SAP and UAP patients are explored, meanwhile linkage with somatic and cognitive depressive symptoms is assessed. Our findings reveal that depression symptoms in CHD patients are actually to a large extend derived from the disease itself and exacerbate along with the deterioration of physical status especially when CHD is unstable. Discomfort, as the reason leading to the increment of somatic symptom score, probably at the same time arouses cognitive symptoms. Anxiety symptoms, though generally positively correlate with depression symptoms, may exhibit an inverse relation along with the worsening of physical condition. However, no significant association between NYHA classes and anxiety in the separate analysis was discovered. These findings can at least partly be supported by the phenomenon that left ventricular assist device can help heart failure patients reduce anxiety and depression [48] and antidepressant is hardly to be efficient to improve prognosis in CHD patients [49, 50].

Our findings should be considered in light of several potential limitations. First, due to small sample size, NYHA class IV group of patients could not be investigated separately. Therefore, the present study may be unable to represent the seriously ill classification of NYHA IV. Besides, a small sample size might lead to an inaccurate outcome, especially for the analysis on clinical anxious patients and some variables could therefore not been adjusted. However, it should be noted that most of our findings were obtained based on the same outcomes with two criteria, which makes the conclusion more persuasive. Second, this is a single centered study. The advantage is that we could minimize the measuring error by fixing the tester. The disadvantage is that generalizability of the study results needs careful consideration. Third, our data were collected mainly based on the status of patients at admission. Even though all patients were warranted to be surveyed in comparatively stable state, the acute phase of disease was still possible to interfere the assessment results. Lastly, due to the limitation of linear model, we could only from several viewpoints to speculate the complicated interactive relationship between CHD and mood symptoms. More complex model is needed to reveal the deeper associations.

## Conclusions

In summary, our study demonstrated a high synchronized alteration of somatic and cognitive depressive symptoms along with the progress of disease severity. However, more intense mood symptoms are prone to be aroused when patients are in bad functional status. Education background has greater impact on mood when patient’s condition is unstable. These findings may trigger deeper rethink of the associations of mood symptoms with CHD and with the prognosis, lead to a better understanding of the mechanism of mood disorder in CHD patients and help to make the intervention more timely and efficient.

## Additional files

Additional file 1: **Table S1.** Comparison of fit statistics for the five previously hypothesized factor models of PHQ-9. (DOCX 16 kb)

Additional file 2: **Table S2.** Characteristics of patients stratified by anxiety severity. (DOCX 22 kb)

Additional file 3: **Table S3.** Association between NYHA classes and clinical features using multivariate ordinal logistic regression model. (DOCX 17 kb)

## Abbreviations

ACEI: angiotensin converting enzyme inhibitor
AMI: acute myocardial infarction
ANOVA: one-way analysis of variance
AP: angina pectoris
ARB: angiotensin receptor blocker
BMI: body mass index
CAG: coronary angiography
CFA: confirmatory factor analysis
CHD: coronary heart disease
GAD: Generalized Anxiety Disorder Scale
LVEF: left ventricular ejection fraction
NYHA class: New York Heart Association functional class
PCI: percutaneous transluminal coronary intervention
PHQ: Patient Health Questionnaire
SAP: stable angina pectoris
UAP: unstable angina pectoris
