# Supplementary material for: Associations of mood symptoms with NYHA functional classes in angina pectoris patients: a cross-sectional study
Source: BMC Psychiatry. 2019 Mar 5;19:85. doi: 10.1186/s12888-019-2061-3 (PMC6402172; doi:10.1186/s12888-019-2061-3)
Supplement: Supplementary file 1 — Table S1. Comparison of fit statistics for the five previously hypothesized factor models of PHQ-9. (DOCX 16 kb) [file 12888_2019_2061_MOESM1_ESM.docx]

| **Additional file 1: Table S1. Comparison of fit statistics for the five previously hypothesized factor models of PHQ-9** | | | | | |
| --- | --- | --- | --- | --- | --- |
|  |  |  |  |  |  |
| PHQ 9 items | Model 1 | Model 2a | Model 2b | **Model 2c** | Model 2d |
| 1 Anhedonia | Depression | Cognitive | Cognitive | **Cognitive** | Somatic |
| 2 Depressed mood | Depression | Cognitive | Cognitive | **Cognitive** | Cognitive |
| 3 Sleep difficulties | Depression | Somatic | Somatic | **Somatic** | Somatic |
| 4 Fatigue | Depression | Somatic | Somatic | **Somatic** | Somatic |
| 5 Appetite changes | Depression | Somatic | Somatic | **Somatic** | Somatic |
| 6 Feeling of worthlessness | Depression | Cognitive | Cognitive | **Cognitive** | Cognitive |
| 7 Concentration difficulties | Depression | Cognitive | Somatic | **Somatic** | Somatic |
| 8 Psychomotor agitation/retardation | Depression | Cognitive | Cognitive | **Somatic** | Somatic |
| 9 Thoughts of death | Depression | Cognitive | Cognitive | **Cognitive** | Cognitive |
| Fit indices | Confirmatory factor analyses | | | | |
| Satorra-Bentler χ^2^ | 95.735 | 78.250 | 82.914 | **74.270** | 79.380 |
| d.f. | 27 | 26 | 26 | **26** | 26 |
| CFI | 0.898 | 0.922 | 0.911 | **0.928** | 0.921 |
| TLI | 0.864 | 0.892 | 0.883 | **0.900** | 0.890 |
| RMSEA | 0.076 | 0.067 | 0.070 | **0.065** | 0.068 |
| SRMR | 0.051 | 0.045 | 0.047 | **0.045** | 0.046 |
| Abbreviation: d.f.: degree of freedom; CFI: Comparative fit index; TLI: Tucker–Lewis Index; RMSEA: Root mean square error of approximation; SRMR: standardized root mean square residual. | | | | | |
|  |  |  |  |  |  |
