# Supplementary material for: Associations of mood symptoms with NYHA functional classes in angina pectoris patients: a cross-sectional study
Source: BMC Psychiatry. 2019 Mar 5;19:85. doi: 10.1186/s12888-019-2061-3 (PMC6402172; doi:10.1186/s12888-019-2061-3)
Supplement: Supplementary file 2 — Table S2. Characteristics of patients stratified by anxiety severity. (DOCX 22 kb) [file 12888_2019_2061_MOESM2_ESM.docx]

| **Additional file 2: Table S2. Characteristics of patients stratified by anxiety severity** | | | | | | | | | |
| --- | --- | --- | --- | --- | --- | --- | --- | --- | --- |
| **Variables** | **Total** | **Non- anxious** | **Anxious** | | | ***p* value** | | ***p* value** | |
|  |  |  | **mild anx.** | **mod- severe anx.** | |  |  |  |  |
|  | N=443 | N=316 | N=103 (5≤score<10) | | N=24 | | not clinical  vs clinical | | nonanxious  vs anxious |
|  |  | 71.3% (score<5) | 23.3% | | 5.4% (score≥10) | |  |  |  |
| **Demographics** |  |  |  | |  | |  | |  |
| Age,mean±SD,y | 63.9±9.8 | 64.2±9.9 | 63.4±8.9 | | 62.5±12.7 | | 0.57 | | 0.36 |
| Male ,No.(%) | 337(76.1) | 256(81.0) | 63(61.2) | | 18(75) | | 0.90 | | <.001 |
| Body mass index,mean±SD,kg/m^2^ | 24.5±3.1 | 24.5±3.0 | 24.3±3.1 | | 25.4±4.1 | | 0.29 | | >0.99 |
| **Clinical characteristics** |  |  |  | |  | |  | |  |
| NYHA class I-IV,No.(%) |  |  |  | |  | | 0.37 | | 0.77 |
| Class I | 115(26.0) | 80(25.3) | 30(29.1) | | 5(20.8) | |  | |  |
| Class II | 261(58.9) | 186(58.9) | 62(60.2) | | 13(54.2) | |  | |  |
| Class III-IV | 67(15.1) | 50(15.8) | 11(10.7) | | 6(25) | |  | |  |
| Ejection Fraction,mean±SD,% | 59.3±11.0 | 59.0±11.4 N=284 | 60.2±9.8 N=103 | | 58.4±11.4 N=20 | | 0.72 | | 0.52 |
| Nt-ProBNP,median(interquartile range),pg/mL | 123(50-382) | 125(54-382) N=262 | 119(32-309) N=88 | | 124(60-573) N=19 | | 0.75 | | 0.19 |
| Creatinine Clearance,mean±SD,ml/min | 65.5±21.7 | 65.6±21.1 | 65.5±20.5 | | 64.3±32.8 | | 0.85 | | 0.88 |
| Type of angina pectoris,No.(%) |  |  |  | |  | | 0.37 | | 0.23 |
| Unstable angina pectoris | 256(57.8) | 177(56.0) | 63(61.2) | | 16(66.7) | |  | |  |
| Stable angina pectoris | 187(42.2) | 139(44.0) | 40(38.8) | | 8(33.3) | |  | |  |
| Severity of coronary stenosis,No.(%) |  |  |  | |  | | 0.52 | | .050 |
| 1 | 93(21.0) | 56(17.7) | 32(31.1) | | 5(20.8) | |  | |  |
| 2 | 82(18.5) | 64(20.3) | 11(10.7) | | 7(29.2) | |  | |  |
| 3 | 268(60.5) | 196(62.0) | 60(58.3) | | 12(50.0) | |  | |  |
| **Socialeconomic factors** |  |  |  | |  | |  | |  |
| Education,No.(%) |  |  |  | |  | | 0.57 | | .001 |
| less than 6 years | 115(26.0) | 67(21.2) | 38(36.9) | | 10(41.7) | |  | |  |
| 7-9 years | 126(28.4) | 93(29.4) | 28(27.2) | | 5(20.8) | |  | |  |
| 10-12 years | 97(21.9) | 70(22.2) | 23(22.3) | | 4(16.7) | |  | |  |
| more than 12 years | 105(23.7) | 86(27.2) | 14(13.6) | | 5(20.8) | |  | |  |
| Marriage,No.(%) |  |  |  | |  | | 0.79 | | 0.96 |
| Married | 412(93.0) | 294(93.0) | 96(93.2) | | 22(91.7) | |  | |  |
| Divorced or Widowed or Single | 31(1.6) | 22(7.0) | 7(6.8) | | 2(8.3) | |  | |  |
| **Medical history,No.(%)** |  |  |  | |  | |  | |  |
| Hypertension | 276(62.3) | 197(62.3) | 61(59.2) | | 16(66.7) | | 0.62 | | 0.74 |
| Diabetes mellitus | 154(34.8) | 114(36.1) | 31(30.1) | | 9(37.5) | | 0.77 | | 0.36 |
| Prior PCI | 167(37.7) | 123(38.9) | 36(35.0) | | 10(41.7) | | 0.72 | | 0.60 |
| History of antidepressant treatment | 17(3.8) | 5(1.6) | 8(7.7) | | 4(16.7) | | .010 | | <.001 |
| **Medication use,No.(%)** |  |  |  | |  | |  | |  |
| ACEI or ARB | 317(71.6) | 233(73.7) | 69(67.0) | | 15(62.5) | | 0.31 | | 0.11 |
| β blocker | 384(86.7) | 275(87.0) | 86(83.5) | | 23(95.8) | | 0.17 | | 0.74 |
| Mono antipletelet therapy | 63(14.2) | 44(13.9) | 16(15.5) | | 4(16.7) | | 0.98 | | 0.62 |
| Dual antiplatelet therapy | 370(83.5) | 266(84.2) | 83(80.6) | | 20(83.3) | | >0.99 | | 0.43 |
| Statin | 430(97.1) | 310(98.1) | 99(96.1) | | 24(100) | | # | | 0.42 |
| Aldosterone receptor antagonist | 42(9.5) | 29(9.3) | 10(9.7) | | 3(12.5) | | 0.87 | | 0.73 |
| Loop diuretic | 47(10.6) | 32(10.1) | 11(10.4) | | 4(16.7) | | 0.52 | | 0.60 |
| Anticoagulant | 20(4.5) | 12(3.8) | 8(7.8) | | 0(0) | | # | | 0.25 |
| Antidepressant | 9(2.0) | 5(1.6) | 3(2.9) | | 1(4.2) | | 0.40 | | 0.49 |
| **Note: Clinical characteristics were compared between subjects with GAD-7 score <10 and ≥10 (not clinical vs clinical) as well as <5 and ≥5 (non-anxious vs anxious).** | | | | | | | | | |
| #: chi-square test did not fit. | | | | | | | | | |
| Abbreviation: anx.: anxiety; mod-severe anx.: moderate or severe anxiety; PCI: percutaneous transluminal coronary intervention; ACEI: angiotensin converting enzyme inhibitor; ARB: angiotensin receptor blocker. | | | | | | | | | |
