# Supplementary material for: Associations of mood symptoms with NYHA functional classes in angina pectoris patients: a cross-sectional study
Source: BMC Psychiatry. 2019 Mar 5;19:85. doi: 10.1186/s12888-019-2061-3 (PMC6402172; doi:10.1186/s12888-019-2061-3)
Supplement: Supplementary file 3 — Table S3. Association between NYHA classes and clinical features using multivariate ordinal logistic regression model. (DOCX 17 kb) [file 12888_2019_2061_MOESM3_ESM.docx]

| **Additional file 3: Table S3 Association between NYHA classes and clinical features using multivariate ordinal logistic regression model.** | | |
| --- | --- | --- |
|  | **Multivariate-adjusted model** | ***p* value** |
|  | **Odds Ratio (95% CI)** |  |
| **Age (per 1 year increase)** | **1.04(1.01,1.08)** | **.024** |
| Sex |  |  |
| female vs male | 0.61(0.34,1.10) | 0.10 |
| Body mass index (per 1 kg/m^2^ increase) | 1.06(0.97,1.17) | 0.19 |
| Education |  | 0.59 |
| less than 6 years | reference |  |
| 7-9 years vs less than 6 years | 0.78(0.41,1.47) | 0.44 |
| 10-12 years vs less than 6 years | 0.72(0.36,1.42) | 0.34 |
| more then 12 years vs less than 6 years | 0.62(0.31,1.24) | 0.18 |
| **Ejection Fraction (per 1% increase)** | **0.97(0.95,1.00)** | **.037** |
| **Nt-ProBNP** |  | **.006** |
| first quartile | reference |  |
| second quartile vs first quartile | 1.37(0.70,2.68) | 0.37 |
| third quartile vs first quartile | 0.77(0.39,1.52) | 0.46 |
| fourth quartile vs first quartile | 2.78(1.23,6.32) | .015 |
| Creatinine Clearance (per 1ml/min increase) | 1.00(0.98,1.02) | 0.90 |
| **Severity of coronary artery stenosis** | **1.37(1.03,1.84)** | **.034** |
| Hypertension | 1.29(0.80,2.09) | 0.30 |
| **Diabetes mellitus** | **1.85(1.12,3.05)** | **.016** |
| **Note:** Model treated NYHA classes I, II, III/IV as ordinal outcomes and was adjusted for age, sex, education, BMI, EF, Nt-ProBNP, CCR, severity of coronary stenosis, history of hypertension and diabetes. | | |
